# Complete Genome Sequence of Streptococcus suis Serotype 2 Virulent Strain SS2-1

**DOI:** 10.1128/genomeA.00067-18

**Published:** 2018-03-08

**Authors:** Haodan Zhu, Yanxiu Ni, Junming Zhou, Zhengyu Yu, Aihua Mao, Dandan Wang, Kongwang He

**Affiliations:** a Institute of Veterinary Medicine, Jiangsu Academy of Agricultural Sciences, Nanjing, Jiangsu, China; b Jiangsu Co-innovation Center for Prevention and Control of Important Animal Infectious Diseases and Zoonoses, Yangzhou, Jiangsu, China

## Abstract

Streptococcus suis is an important swine pathogen that can also cause severe diseases in humans. Herein, we describe the genome sequence of Streptococcus suis serotype 2 virulent strain SS2-1, which was isolated from a diseased dead pig amid the 1998 Streptococcus suis outbreak in Jiangsu Province in China.

## GENOME ANNOUNCEMENT

Streptococcus suis is a very important Gram-positive bacterium considered worldwide to be one of the most important pathogens in the swine industry. S. suis causes a wide variety of diseases in pigs, including meningitis, septicemia, and endocarditis (1, 2). S. suis is also an emerging zoonotic agent responsible for septicemia with or without septic shock, meningitis, and other less common infections in humans, particularly in Asian countries (3). Among the 33 known serotypes, serotype 2 (SS2) is considered to be the most prevalent and virulent in pigs and humans (4, 5). Two large-scale outbreaks of SS2 in China in 1998 and in 2005 have posed public health concerns worldwide (6).

The SS2 virulent strain SS2-1 was isolated from a diseased dead pig with septicemia in Jiangsu province in 1998 and has been confirmed as virulent on the basis of animal experiments (7). The complete genome sequence was determined by the Illumina HiSeq platform at Novogene (Beijing, China). Assembly was performed using SOAPdenovo. Gaps were filled by primer walking and sequencing of PCR products. The assembly of the genome was further verified by PCR. Coding sequences (CDS) were predicted using Glimmer 3.02 and GeneMarkS (8) and further examined with the nonredundant protein database through BLASTp. tRNAs and rRNAs were identified using tRNAscan-SE and RNAmmer (9), respectively.

The genome of strain SS2-1 consists of a single circular chromosome which is 2,094030 bp in length, with a GC content of 41.10%. There are 1,989 CDS that account for 94.5% of the genome (2,105 genes), 47 tRNAs, 11 rRNAs, 4 noncoding RNAs, and 54 pseudogenes. The genome of SS2-1 was found to be in an orientation similar to that of the majority of the published genomes of SS2 strains in GenBank (10). The genome of SS2-1 harbors some virulence gene candidates, such as *mrp*, *epf*, *sly*, *pgdA*, *DltA*, *srtA*, *salK*/*salR*, *nisK*/*nisR*, *ssnA*, *sspA*, *ideSsuis*, *ssadS*, and *STK*/*STP*, and an 89-kb pathogenicity island (89K PAI) (11, 12). The genomic features of SS2-1 will support the analysis of comparative genomic and pathogenic characteristics of S. suis and development of therapeutic agents and vaccines, which will ultimately help protect the swine industry and human health (13).

### Accession number(s).

The complete genome sequence of S. suis serotype 2 virulent strain SS2-1 has been assigned GenBank accession number CP018908.
